# Recurrent Neural Networks for Multivariate Time Series with Missing Values

**DOI:** 10.1038/s41598-018-24271-9

**Published:** 2018-04-17

**Authors:** Zhengping Che, Sanjay Purushotham, Kyunghyun Cho, David Sontag, Yan Liu

**Affiliations:** 1 University of Southern California, Department of Computer Science, Los Angeles, CA 90089 USA; 2 New York University, Department of Computer Science, New York, NY 10012 USA; 3 Massachusetts Institute of Technology, Department of Electrical Engineering and Computer Science, Cambridge, MA 02139 USA

## Abstract

Multivariate time series data in practical applications, such as health care, geoscience, and biology, are characterized by a variety of missing values. In time series prediction and other related tasks, it has been noted that missing values and their missing patterns are often correlated with the target labels, a.k.a., *informative* missingness. There is very limited work on exploiting the missing patterns for effective imputation and improving prediction performance. In this paper, we develop novel deep learning models, namely **GRU**-**D**, as one of the early attempts. GRU-D is based on Gated Recurrent Unit (GRU), a state-of-the-art recurrent neural network. It takes two representations of missing patterns, i.e., *masking* and *time interval*, and effectively incorporates them into a deep model architecture so that it not only captures the long-term temporal dependencies in time series, but also utilizes the missing patterns to achieve better prediction results. Experiments of time series classification tasks on real-world clinical datasets (MIMIC-III, PhysioNet) and synthetic datasets demonstrate that our models achieve state-of-the-art performance and provide useful insights for better understanding and utilization of missing values in time series analysis.

## Introduction

**Figure 1:**
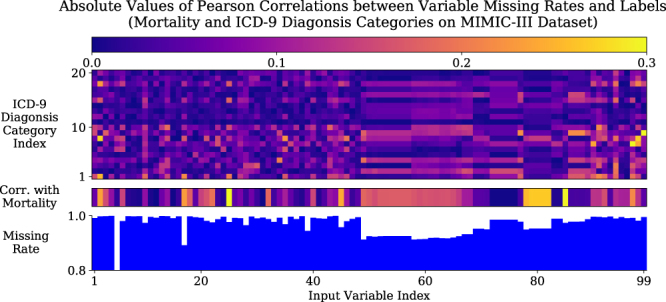
Demonstration of informative missingness on MIMIC-III dataset. The bottom figure shows the missing rate of each input variable. The middle figure shows the absolute values of Pearson correlation coefficients between missing rate of each variable and mortality. The top figure shows the absolute values of Pearson correlation coefficients between missing rate of each variable and each ICD-9 diagnosis category. More details can be found in supplementary information Section S1.

Multivariate time series data are ubiquitous in many practical applications ranging from health care, geoscience, astronomy, to biology and others. They often inevitably carry missing observations due to various reasons, such as medical events, saving costs, anomalies, inconvenience and so on. It has been noted that these missing values are usually *informative missingness*^1^, i.e., the missing values and patterns provide rich information about target labels in supervised learning tasks (e.g, time series classification). To illustrate this idea, we show some examples from MIMIC-III^2^, a real world health care dataset, in Fig. 1. We plot the Pearson correlation coefficient between variable missing rates, which indicates how often the variable is missing in the time series, and the labels of our interests, which are mortality and ICD-9 diagnosis categories. We observe that the value of missing rate is correlated with the labels, and the missing rate of variables with low missing rate are usually highly (either positive or negative) correlated with the labels. In other words, the missing rate of variables for each patient is useful, and this information is more useful for the variables which are observed more often in the dataset. These findings demonstrate the usefulness of missingness patterns in solving a prediction task.

In the past decades, various approaches have been developed to address missing values in time series^3^. A simple solution is to omit the missing data and to perform analysis only on the observed data, but it does not provide good performance when the missing rate is high and inadequate samples are kept. Another solution is to fill in the missing values with substituted values, which is known as data imputation. Smoothing, interpolation^4^, and spline^5^ methods are simple and efficient, thus widely applied in practice. However, these methods do not capture variable correlations and may not capture complex pattern to perform imputation. A variety of imputation methods have been developed to better estimate missing data. These include spectral analysis^6^, kernel methods^7^, EM algorithm^8^, matrix completion^9^ and matrix factorization^10^. Multiple imputation^11,12^ can be further applied with these imputation methods to reduce the uncertainty, by repeating the imputation procedure multiple times and averaging the results. Combining the imputation methods with prediction models often results in a two-step process where imputation and prediction models are separated. By doing this, the missing patterns are not effectively explored in the prediction model, thus leading to suboptimal analyses results^13^. In addition, most imputation methods also have other requirements which may not be satisfied in real applications, for example, many of them work on data with small missing rates only, assume the data is missing at random or completely at random, or can not handle time series data with varying lengths. Moreover, training and applying these imputation methods are usually computationally expensive.

Recently, Recurrent Neural Networks (RNNs), such as Long Short-Term Memory (LSTM)^14^ and Gated Recurrent Unit (GRU)^15^, have shown to achieve the state-of-the-art results in many applications with time series or sequential data, including machine translation^16,17^ and speech recognition^18^. RNNs enjoy several nice properties such as strong prediction performance as well as the ability to capture long-term temporal dependencies and variable-length observations. RNNs for missing data have been studied in earlier works^19–21^ and applied for speech recognition and blood-glucose prediction. Recent works^22,23^ tried to handle missingness in RNNs by concatenating missing entries or timestamps with the input or performing simple imputations. However, there have not been works which design RNN structures incorporating the patterns of missingness for time series classification problems. Exploiting the power of customized RNN models along with the *informativeness* of missing patterns is a new promising venue to effectively model multivariate time series and is the main motivation behind our work.

In this paper, we develop a novel deep learning model based on GRU, namely GRU-D, to effectively exploit two representations of informative missingness patterns, i.e., *masking* and *time interval*. Masking informs the model which inputs are observed (or missing), while time interval encapsulates the input observation patterns. Our model captures the observations and their dependencies by applying masking and time interval (using a decay term) to the inputs and network states of GRU, and jointly train all model components using back-propagation. Thus, our model not only captures the long-term temporal dependencies of time series observations but also utilizes the missing patterns to improve the prediction results. Empirical experiments on real-world clinical datasets as well as synthetic datasets demonstrate that our proposed model outperforms strong deep learning models built on GRU with imputation as well as other strong baselines. These experiments show that our proposed method is suitable for many time series classification problems with missing data, and in particular is readily applicable to the predictive tasks in emerging health care applications. Moreover, our method provides useful insights into more general research challenges of time series analysis with missing data beyond classification tasks, including 1) a general deep learning framework to handle time series with missing data, 2) an effective solution to characterize the missing patterns of not missing-completely-at-random time series data with masking and time interval, and 3) an insightful approach to study the impact of variable missingness on the prediction labels by decay analysis.

## Methods

### Notations

**Figure 2:**
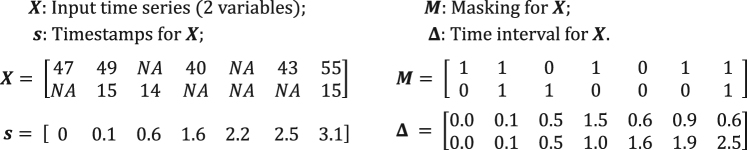
An example of measurement vectors *x*_*t*_, time stamps *s*_*t*_, masking *m*_*t*_, and time interval *δ*_*t*_.

We denote a multivariate time series with *D* variables of length *T* as $X={({x}_{1},{x}_{2},\ldots ,{x}_{T})}^{{\rm{{\rm T}}}}\in {{\mathbb{R}}}^{T\times D}$, where for each *t* ∈ {1, 2, …, *T*}, ${x}_{t}\in {{\mathbb{R}}}^{D}$ represents the *t*-th observations (a.k.a., measurements) of all variables and ${x}_{t}^{d}$ denotes the measurement of *d*-th variable of *x*_*t*_. Let ${s}_{t}\in {\mathbb{R}}$ denote the time-stamp when the *t*th observation is obtained and we assume that the first observation is made at time-stamp 0 (i.e., *s*_1_ = 0). A time series *X* could have missing values. We introduce a *masking vector m*_*t*_ ∈ {0, 1}^*D*^ to denote which variables are missing at time step *t*, and also maintain the *time interval* ${\delta }_{t}^{d}\in {\mathbb{R}}$ for each variable *d* since its last observation. To be more specific, we have

1 $${m}_{t}^{d}=\{\begin{array}{ll}1, & {\rm{if}}\,{x}_{t}^{d}\,{\rm{is}}\,{\rm{observed}}\\ 0, & {\rm{otherwise}}\end{array}$$

2 $${\delta }_{t}^{d}=\{\begin{array}{ll}{s}_{t}-{s}_{t-1}+{\delta }_{t-1}^{d}, & t > 1,{m}_{t-1}^{d}=0\\ {s}_{t}-{s}_{t-1}, & t > 1,{m}_{t-1}^{d}=1\\ \mathrm{0,} & t=1\end{array}$$

and an example of the notations is illustrated in Fig. 2. In this paper, we are interested in the time series classification problem, where we predict the labels *l*_*n*_ ∈ {1, …, *L*} given the time series data ${\mathscr{D}}$, where ${\mathscr{D}}={\{({X}_{n},{s}_{n},{M}_{n})\}}_{n=1}^{N}$, and ${X}_{n}=[{x}_{1}^{(n)},\ldots ,{x}_{{T}_{n}}^{(n)}]$, ${s}_{n}=[{s}_{1}^{(n)},\ldots ,{s}_{{T}_{n}}^{(n)}]$, ${M}_{n}=[{m}_{1}^{(n)},\ldots ,{m}_{{T}_{n}}^{(n)}]$.

### GRU-RNN for time series classification

We investigate the use of recurrent neural networks (RNN) for time-series classification, as their recursive formulation allows them to handle variable-length sequences naturally. Moreover, RNN shares the same parameters across all time steps, which greatly reduces the total number of parameters we need to learn. Among different variants of the RNN, we specifically consider an RNN with gated recurrent units (GRUs)^15^, but similar discussions and modifications are also valid for other RNN models such as Long Short-Term Memory (LSTM)^14^.

**Figure 3:**
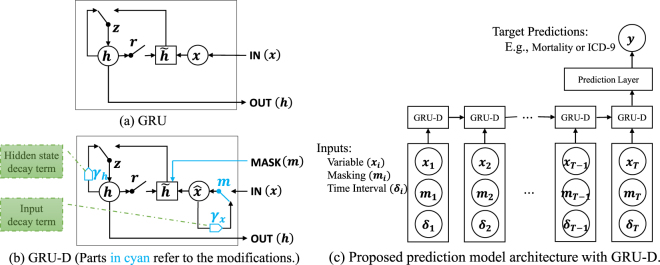
Graphical illustrations of the original GRU (top-left), the proposed GRU-D (bottom-left), and the whole network architecture (right).

The structure of GRU is shown in Fig. 3(a). For each *j*-th hidden unit, GRU has a reset gate ${r}_{t}^{j}$ and an update gate ${z}_{t}^{j}$ to control the hidden state ${h}_{t}^{j}$ at each time *t*. The update functions are as follows:

3 $${r}_{t}=\sigma ({W}_{r}{x}_{t}+{U}_{r}{h}_{t-1}+{b}_{r})$$

4 $${z}_{t}=\sigma ({W}_{z}{x}_{t}+{U}_{z}{h}_{t-1}+{b}_{z})$$

5 $${\tilde{h}}_{t}=\,\tanh (W{x}_{t}+U({r}_{t}\odot {h}_{t-1})+b)$$

6 $${h}_{t}=(1-{z}_{t})\odot {h}_{t-1}+{z}_{t}\odot {\tilde{h}}_{t}$$

where matrices *W*_*z*_, *W*_*r*_, *W*, *U*_*z*_, *U*_*r*_, *U* and vectors *b*_*z*_, *b*_*r*_, *b* are model parameters. We use *σ* for element-wise sigmoid function, and $\odot$ for element-wise multiplication. This formulation assumes that all the variables are observed. A sigmoid or soft-max layer is then applied on the output of the GRU layer at the last time step for classification task.

There are three straightforward ways to handle missing values without applying any imputation approaches or making any modifications to GRU network architecture. The first approach is simply to replace each missing observation with the mean of the variable across the training examples. In the context of GRU, we have

7 $${x}_{t}^{d}\leftarrow {m}_{t}^{d}{x}_{t}^{d}+\mathrm{(1}-{m}_{t}^{d}){\tilde{x}}^{d}$$

where ${\tilde{x}}^{d}={\sum }_{n=1}^{N}\,{\sum }_{t=1}^{{T}_{n}}\,{m}_{t,n}^{d}{x}_{t,n}^{d}/{\sum }_{n=1}^{N}\,{\sum }_{t=1}^{{T}_{n}}\,{m}_{t,n}^{d}$. ${\tilde{x}}^{d}$ is calculated on the training dataset and used for both training and testing datasets. We refer to this approach as **GRU**-**Mean**.

A second approach is to exploit the temporal structure. For example, we may assume any missing value is the same as its last measurement and use forward imputation (**GRU**-**Forward**), i.e.,

8 $${x}_{t}^{d}\leftarrow {m}_{t}^{d}{x}_{t}^{d}+\mathrm{(1}-{m}_{t}^{d}){x}_{t^{\prime} }^{d}$$

where *t*′ < *t* is the last time the *d*-th variable was observed.

Instead of explicitly imputing missing values, the third approach simply indicates which variables are missing and how long they have been missing as a part of input by concatenating the measurement, masking and time interval vectors as

9 $${x}_{t}^{(n)}\leftarrow [{x}_{t}^{(n)};{m}_{t}^{(n)};{\delta }_{t}^{(n)}]$$

where ${x}_{t}^{(n)}$ can be either from Equation (7) or (8). We later refer to this approach as **GRU**-**Simple**.

Several recent works^22–24^ use RNNs on EHR data to model diseases and to predict patient diagnosis from health care time series data with irregular time stamps or missing values, but none of them have explicitly attempted to capture and utilize the missing patterns into their RNNs via systematically modified network architectures. Also it’s worth noting that although we focus on the same topic of handling missing values in time series by RNN, our work^25^ was done independently of any of these.

One model^23^ feeds medical codes along with its time stamps into GRU model to predict the next medical event. This idea of feeding time stamps along with the input values is equivalent to the baseline GRU-Simple with only the interval but not the masking (*m*), which we denote as **GRU**-**Simple w/o** *m*. Another model^24^ uses LSTM model and extends the forget gate in LSTM to a logarithmic or cubic decay function of time intervals between two time stamps. Their model is essentially similar to GRU-Simple w/o *m*. Neither of them consider missing values in time series medical records. In addition, they only take one time stamp sequence for all variables. Unlike these two models, our model keep track of the time stamps at which measurements were made for each variable separately and thus can be more precise. In another work^22^, the authors achieve their best performance on diagnosis prediction by feeding masking with zero-filled missing values in the recurrent neural network. Their model is equivalent to the GRU-Simple model without feeding the time interval (*δ*) given that the input features are normalized to have mean value 0 before fed into the RNN model. We denote it as **GRU**-**Simple w/o** *δ*. Conclusively, none of the related works mentioned above modify the RNN model structure to further capture and utilize missingness, and our GRU-Simple baseline can be considered as a generalization of all these related RNN models.

These approaches solve the missing value issue to a certain extent, however, imputing the missing value with mean or forward imputation cannot distinguish whether missing values are imputed or truly observed. Simply concatenating masking and time interval vectors fails to exploit the temporal structure of missing values. Thus none of them fully utilize missingness in data to achieve desirable performance.

### GRU-D: model with trainable decays

To fundamentally address the issue of missing values in time series, we notice two important properties of the missing values in time series, especially in the healthcare domain: First, the value of the missing variable tend to be close to some default value if its last observation happens a long time ago. This property usually exists in health care data for human body as homeostasis mechanisms and is considered to be critical for disease diagnosis and treatment^26^. Second, the influence of the input variables will fade away over time if the variable has been missing for a while. For example, one medical feature in electronic health records (EHRs) is only significant in a certain temporal context^27^. Therefore, we propose a GRU-based model called **GRU**-**D** shown in Fig. 3(b), in which a *decay* mechanism is designed for the input variables and the hidden states to capture the aforementioned properties. We introduce *decay rates* in the model to control the decay mechanism by considering the following important factors. First, each input variable in health care time series has its own meaning and importance in medical applications. The decay rates should differ from variable to variable based on the underlying properties associated with the variables. Second, as we see lots of missing patterns are informative and potentially useful in prediction tasks but unknown and possibly complex, we aim at learning decay rates from the training data rather than fixed a priori. That is, we model a vector of decay rates *γ* as

10 $${\gamma }_{t}=\exp \{\,-\,{\rm{\max }}(\mathrm{0,}{W}_{\gamma }{\delta }_{t}+{b}_{\gamma })\}$$

where *W*_*γ*_ and *b*_*γ*_ are model parameters that we train jointly with all the other parameters of the GRU. We chose the exponentiated negative rectifier in order to keep each decay rate monotonically decreasing in a reasonable range between 0 and 1. Note that other formulations such as a sigmoid function can be used instead, as long as the resulting decay is monotonic and is in the same range.

Our proposed **GRU**-**D** model incorporates two different trainable decay mechanisms to utilize the missingness directly with the input feature values and implicitly in the RNN states. First, for a missing variable, we use an *input decay γ*_*x*_ to decay it over time toward the empirical mean (which we take as a *default* configuration), instead of using the last observation as it is. Under this assumption, the trainable decay scheme can be readily applied to the measurement vector by

11 $${\hat{x}}_{t}^{d}={m}_{t}^{d}{x}_{t}^{d}+(1-{m}_{t}^{d})\,({\gamma }_{{x}_{t}}^{d}{x}_{t^{\prime} }^{d}+\mathrm{(1}-{\gamma }_{{x}_{t}}^{d}){\tilde{x}}^{d})$$

where ${x}_{t^{\prime} }^{d}$ is the last observation of the *d*-th variable (*t*′ < *t*) and ${\tilde{x}}^{d}$ is the empirical mean of the *d*-th variable. When decaying the input variable directly, we constrain ${W}_{{\gamma }_{x}}$ to be diagonal, which effectively makes the decay rate of each variable independent from the others.

Sometimes the input decay may not fully capture the missing patterns since not all missingness information can be represented in decayed input values. In order to capture richer knowledge from missingness, we also have a *hidden state decay γ*_*h*_ in GRU-D. Intuitively, this has an effect of decaying the extracted features (GRU hidden states) rather than raw input variables directly. This is implemented by decaying the previous hidden state *h*_*t*−1_ before computing the new hidden state *h*_*t*_ as

12 $${\hat{h}}_{t-1}={\gamma }_{{h}_{t}}\odot {h}_{t-1},$$

in which case we do not constrain ${W}_{{\gamma }_{h}}$ to be diagonal. In addition, we feed the masking vectors (*m*_*t*_) directly into the model. The update functions of GRU-D are

13 $${r}_{t}=\sigma ({W}_{r}{\hat{x}}_{t}+{U}_{r}{\hat{h}}_{t-1}+{V}_{r}{m}_{t}+{b}_{r})$$

14 $${z}_{t}=\sigma ({W}_{z}{\hat{x}}_{t}+{U}_{z}{\hat{h}}_{t-1}+{V}_{z}{m}_{t}+{b}_{z})$$

15 $${\tilde{h}}_{t}=\,\tanh (W{\hat{x}}_{t}+U({r}_{t}\odot {\hat{h}}_{t-1})+V{m}_{t}+b)$$

16 $${h}_{t}=(1-{z}_{t})\odot {\hat{h}}_{t-1}+{z}_{t}\odot {\tilde{h}}_{t}$$

It is worth noting that the main differences between formulas of GRU-D in Equations (13) to (16) and those of standard GRU in Equations (3) to (6). First, *x*_*t*_ and *h*_*t*−1_ are respectively replaced by ${\hat{x}}_{t}$ and ${\hat{h}}_{t-1}$ from Equations (11) and (12). Second, the masking vector *m*_*t*_ are fed into the model, and *V*_*z*_, *V*_*r*_, *V* are new parameters for it.

In our final prediction model, we use the proposed GRU-D component at each time step, and apply a fully connected prediction layer, which has sigmoid activation for binary classification task or soft-max activation for multi-class classification tasks, on top of the last GRU component. The network architecture is shown in Fig. 3(c). For all datasets in our experiments, the same network structure is used with different settings on network size including the input, hidden state and output dimensions and the temporal lengths. Several model variations based on GRU-D are discussed in supplementary information Section S2. The idea of decay term can be generalized to LSTM straightforwardly, and it can be generalized to other domains where time series data come with missing patterns which contain useful information in a variety of ways in practical applications.

### Baseline imputation methods

A common way to solve classification task with missing values is first filling the missing values and then applying predictive models on the imputed data. This usually requires to train additional models with extra running cost, and the imputed data quality can not be guaranteed. Our model avoids to rely on external imputation methods, but to have a fair and complete comparison, we test several interpolation and imputation methods and apply them to other prediction baselines in our experiments.

We include the following interpolation and imputation methods:

- *Mean*, *Forward*, *Simple*: We take the mean-imputation (Mean), forward-imputation (Forward), and concatenating the measurement with masking and time interval (Simple) as three imputation baselines. These strategies are described in the GRU-RNN baseline section and can be performed directly without training any imputation models on all predictive models.
- *SoftImpute*^9^: This method uses matrix completion via iterative soft-thresholded Singular Value Decomposition (SVD) to impute missing values.
- *KNN*^28^: This method uses *k*-nearest neighbor to find similar samples and imputed unobserved data by weighted average of similar observations.
- *CubicSpline*^5^: In this method, we use cubic spline to interpolate each feature at different time steps.
- *MICE*^12^: The Multiple Imputation by Chained Equations (MICE) method is widely used in practice, which uses chain equations to create multiple imputations for variables of different types.
- *MF*^10^: We use matrix factorization (MF) to fill the missing items in the incomplete matrix by factorizing the matrix into two low-rank matrices.
- *PCA*^29^: We impute the missing values with the principal component analysis (PCA) model.
- *MissForest*^30^: This is a non-parametric imputation method which uses random forests trained on the observed values to predict the missing values.

For MICE, MF, and PCA methods, we treat a multi-variate time series $X\in {{\mathbb{R}}}^{T\times D}$ as *T* data samples and impute them independently, so that these methods can be applied to time series with different lengths. However, for SoftImpute and KNN methods, taking each time step as one sample is unaffordable in terms of running time and space. We then treat each time series *X* as one data point in these two imputation methods. Therefore we can not use them on time series with different lengths. We implemented these models in python based on fancyimpute^31^, predictive_imputer^32^, and SciPy^33^ libraries. We followed their original code and paper for hyperparameter setting and tuning strategies.

### Baseline prediction methods and implementation details

We categorize all evaluated prediction models used in our experiments into three groups:

- *Non*-*RNN Baselines* (*Non*-*RNN*): We evaluate logistic regression (LR), support vector machines (SVM) and random forest (RF), which are widely used in health care applications. We used all imputation methods described in previous section to fill in the missing values before using these prediction methods.
- *RNN Baselines* (*RNN*): We take the RNN baselines described before (GRU-Mean, GRU-Forward, GRU-Simple), and LSTM-Mean (LSTM model with mean-imputation on the missing measurements) as RNN baselines. As mentioned before, these models are widely used in existing work^22–24^ on applying RNN on health care time series data with missing values or irregular time stamps. We also test GRU prediction models on the imputed data as well.
- *Proposed Methods* (*Proposed*): This is our proposed GRU-D model.

The non-RNN models cannot directly handle time series of different lengths. We carefully design experiments for them to capture the *informative missingness* as much as possible to have fair comparison with the RNN methods. We regularly sample the time-series data to get a fixed length input and perform all baseline imputation methods to fill in the missing values. For concatenation method (Simple) of the non-RNN methods, we concatenate the masking vector along with the measurements of the regularly sampled time series. On PhysioNet dataset we sample the time series on an hourly basis and propagate measurements forward (or backward) in time to fill gaps, and on MIMIC-III dataset we consider two hourly samples (in the first 48 hours) and do forward (or backward) imputation. Our preliminary experiments showed 2-hourly samples obtains better performance than one-hourly samples for MIMIC-III. We choose Gaussian radial basis function (RBF) kernel for SVM since it performs better than other kernels. We use the scikit-learn^34^ for the non-RNN model implementation and tune the parameters by cross-validation.

For RNN models, we use a one layer RNN to model the sequence unless otherwise stated, and then apply a soft-max regressor on top of the last hidden state *h*_*T*_ to do classification as shown in Fig. 3(c). We use 100 and 64 hidden units in GRU-Mean for MIMIC-III and PhysioNet datasets, respectively. In order to fairly compare the capacity of all GRU-RNN models, we build each model in proper size so they share similar number of parameters, and the model parameter count comparison can be found in Table S4 in supplementary information. In addition, having comparable number of parameters also makes all the prediction models have number of iterations and training time close in the same scale in all the experiments. Batch normalization^35^ and dropout^36^ of rate 0.5 are applied to the top regressor layer. We train all the RNN models with the Adam optimization method^37^ and use early stopping to find the best weights on the validation dataset. All RNN models are implemented with Keras^38^ and Theano^39^ libraries in Python.

All the input variables are normalized to be of 0 mean and 1 standard deviation. We report the results from 5-fold cross validation in terms of area under the ROC curve (AUC score). To further evaluate the proposed models, we also provide more detailed comparisons and evaluations on multilayer RNN models and with different model sizes.

## Results

### Dataset and task descriptions

We demonstrate the performance of our proposed models on one synthetic and two real-world health-care datasets and compare them to several strong machine learning and deep learning approaches in classification tasks. We evaluate our models for different settings such as early prediction and different training sizes and investigate the impact of missing values. Please refer to supplementary information Section S3 for data generation and processing details, lists of extracted features and tasks, and data summaries.

#### Gesture phase segmentation dataset (Gesture)

This UCI dataset^40^ has multivariate time series features, regularly sampled and with no missing values, for 5 different gesticulations. We extracted 378 time series and generate 4 synthetic datasets for the purpose of understanding model behaviors with different missing patterns. We treat it as multi-class classification task. The detailed data generating and preprocessing descriptions can be found in supplementary information Section S3.3.

#### PhysioNet Challenge 2012 dataset (PhysioNet)

This dataset, from *PhysioNet Challenge 2012*^41^, is a publicly available collection of multivariate clinical time series from 8,000 intensive care unit (ICU) records. Each record is a multivariate time series of roughly 48 hours and contains 33 variables such as *Albumin*, *heart*-*rate*, *glucose* etc. The full feature list can be found in supplementary information Section S3.2. We used *Training Set A* subset in our experiments since outcomes (such as in-hospital mortality labels) are publicly available only for this subset. We conduct the following two prediction tasks on this dataset:

- *Mortality task*: Predict whether the patient dies in the hospital. There are 554 patients with positive mortality label. We treat this as a binary classification problem.
- *All 4 tasks*: Predict 4 tasks: in-hospital mortality, length-of-stay less than 3 days, whether the patient had a cardiac condition, and whether the patient was recovering from surgery. We treat this as a multi-task classification problem.

#### MIMIC-III dataset (MIMIC-III)

This public dataset^2^ has deidentified clinical care data with over 58,000 hospital admission records collected at Beth Israel Deaconess Medical Center from 2001 to 2012. We extracted 99 time series features from 19,714 admission records collected during 2008–2012 by Metavision data management system which is still employed at the hospital. We only include patients who are alive in the first 48 hours after admission in our dataset, and only use the first 48 hours data after admission of them. We chose four modalities namely *input events* (fluids into patient, e.g. insulin), *output events* (fluids out of the patient, e.g. urine), *lab events* (lab test results, e.g. pH, Platelet count) and *prescription events* (drugs prescribed by doctors, e.g. aspirin and potassium chloride).

We perform following two predictive tasks:

- *Mortality task*: Predict whether the patient dies in the hospital after 48 hours. There are 1,716 patients with positive mortality label and we perform binary classification.
- *ICD*-*9 Code tasks*: Predict 20 ICD-9 diagnosis categories (e.g., respiratory system diagnosis) for each admission as a multi-task classification problem.

### Data availability

The **Gesture** dataset used in this study is available in the UCI Machine Learning Repository at https://archive.ics.uci.edu/ml/datasets/gesture+phase+segmentation; The **PhysioNet** dataset analysed in this study is available in the PhysioNet website at https://physionet.org/challenge/2012/; The **MIMIC**-**III** dataset analysed in this study is available from MIT Laboratory for Computational Physiology and their collaborating research groups, and this dataset is available upon request at http://dx.doi.org/10.13026/C2XW26.

### Exploiting informative missingness on synthetic datasets

**Figure 4:**
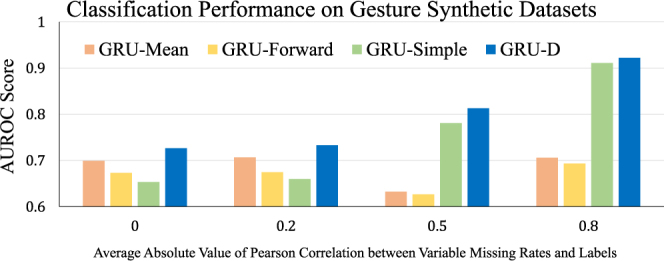
Classification performance on Gesture synthetic datasets with different correlation values.

As illustrated in Fig. 1, missing patterns can be useful in solving prediction tasks. A robust model should exploit informative missingness properly and avoid introducing nonexistent relations between missingness and predictions. To evaluate the impact of modeling missingness we conduct experiments on the synthetic Gesture datasets. We process the data in 4 different settings, with similar missing rate but different correlations between missing rate and the label, which is described in supplementary information. The setting with higher correlation implies more informative missingness. Figure 4 shows the AUC score comparison of three GRU baseline models (GRU-Mean, GRU-Forward, GRU-Simple) and the proposed GRU-D. First, GRU-Mean and GRU-Forward do not utilize any missingness (i.e., masking or time interval) and perform similarly across all 4 settings. GRU-Simple and GRU-D benefit from utilizing the missingness, so they get better performance when the correlation increases. They achieve similar and best performance on the dataset with highest correlation. However, when the correlation is low or non-existent, simply feeding the missingness representations may introduce irrelevant information. As shown in Fig. 4, GRU-Simple fails when the correlation is low. On the other hand, GRU-D has a stable performance and achieves best AUC scores in all the settings. These empirical findings validate our assumption that GRU-D utilizes the missing patterns only when the correlations are high and relies on the observed values when the correlations between labels and missing rates are low. Further, these results on synthetic datasets demonstrate that GRU-D can model the missing patterns properly and does not introduce any non-existent relations.

### Mortality prediction task evaluation on real datasets

We evaluate all methods on MIMIC-III and PhysioNet datasets. We noticed that dropout in the recurrent layer helps a lot for all RNN models on both of the datasets. We apply recurrent dropout^42^ with rate of 0.3 with same dropout samples at each time step on weights *W*, *U*, *V*. Table 1 shows the prediction performance of all the models on mortality task. While using simple imputation methods (Mean, Forward, Simple), all the prediction models except random forest show improved performance when they concatenate missingness indicators along with inputs. The two-step imputation-prediction methods did not improve the prediction performance on these two datasets, and in many cases these methods have worse predictions. This is probably due to the high missing rates in both datasets (>80%) and those imputation methods are not designed for such high missing rates. For example, datasets with a missing rate of 10% to 30% are reported in the related works^30^. Among all these imputation methods, with LR and SVM, the SoftImpute performs the best. CubicSpline, which captures the temporal structure of the data performs the best with RF, but fails with SVM and GRU. MissForest provides slightly better performance with GRU models than other additional imputation baselines. It is worth noting that all these imputation baselines, especially MICE, MF, PCA, and MissForest, generally require a substantial amount of time to train and tune the hyperparameters, thus makes the two-step procedure quite inefficient. Our proposed GRU-D achieves the best AUC score on both the datasets compared with all the other baselines.

**Table 1:** Model performances measured by AUC score (*mean* ± *std*) for mortality prediction.

Non-RNN Models						RNN Models	
*Mortality Prediction On MIMIC*-*III Dataset*						LSTM-Mean	0.8142 ± 0.014
LR-Mean	0.7589 ± 0.015	SVM-Mean	0.7908 ± 0.006	RF-Mean	0.8293 ± 0.004	GRU-Mean	0.8252 ± 0.011
LR-Forward	0.7792 ± 0.018	SVM-Forward	0.8010 ± 0.004	RF-Forward	0.8303 ± 0.003	GRU-Forward	0.8192 ± 0.013
LR-Simple	0.7715 ± 0.015	SVM-Simple	0.8146 ± 0.008	RF-Simple	0.8294 ± 0.007	GRU-Simple w/o *δ*^22^	0.8367 ± 0.009
LR-SoftImpute	0.7598 ± 0.017	SVM-SoftImpute	0.7540 ± 0.012	RF-SoftImpute	0.7855 ± 0.011	GRU-Simple w/o *m*^23,24^	0.8266 ± 0.009
LR-KNN	0.6877 ± 0.011	SVM-KNN	0.7200 ± 0.004	RF-KNN	0.7135 ± 0.015	GRU-Simple	0.8380 ± 0.008
LR-CubicSpline	0.7270 ± 0.005	SVM-CubicSpline	0.6376 ± 0.018	RF-CubicSpline	0.8339 ± 0.007	GRU-CubicSpline	0.8180 ± 0.011
LR-MICE	0.6965 ± 0.019	SVM-MICE	0.7169 ± 0.012	RF-MICE	0.7159 ± 0.005	GRU-MICE	0.7527 ± 0.015
LR-MF	0.7158 ± 0.018	SVM-MF	0.7266 ± 0.017	RF-MF	0.7234 ± 0.011	GRU-MF	0.7843 ± 0.012
LR-PCA	0.7246 ± 0.014	SVM-PCA	0.7235 ± 0.012	RF-PCA	0.7747 ± 0.009	GRU-PCA	0.8236 ± 0.007
LR-MissForest	0.7279 ± 0.016	SVM-MissForest	0.7482 ± 0.016	RF-MissForest	0.7858 ± 0.010	GRU-MissForest	0.8239 ± 0.006
						**Proposed GRU**-**D**	**0.8527 ± 0.003**
*Mortality Prediction On PhysioNet Dataset*						LSTM-Mean	0.8025 ± 0.013
LR-Mean	0.7423 ± 0.011	SVM-Mean	0.8131 ± 0.018	RF-Mean	0.8183 ± 0.015	GRU-Mean	0.8162 ± 0.014
LR-Forward	0.7479 ± 0.012	SVM-Forward	0.8140 ± 0.018	RF-Forward	0.8219 ± 0.017	GRU-Forward	0.8195 ± 0.004
LR-Simple	0.7625 ± 0.004	SVM-Simple	0.8277 ± 0.012	RF-Simple	0.8157 ± 0.014	GRU-Simple	0.8226 ± 0.010
LR-SoftImpute	0.7386 ± 0.007	SVM-SoftImpute	0.8057 ± 0.019	RF-SoftImpute	0.8100 ± 0.016	GRU-SoftImpute	0.8125 ± 0.005
LR-KNN	0.7146 ± 0.011	SVM-KNN	0.7644 ± 0.018	RF-KNN	0.7567 ± 0.012	GRU-KNN	0.8155 ± 0.004
LR-CubicSpline	0.6913 ± 0.022	SVM-CubicSpline	0.6364 ± 0.015	RF-CubicSpline	0.8151 ± 0.015	GRU-CubicSpline	0.7596 ± 0.020
LR-MICE	0.6828 ± 0.015	SVM-MICE	0.7690 ± 0.016	RF-MICE	0.7618 ± 0.007	GRU-MICE	0.8153 ± 0.013
LR-MF	0.6513 ± 0.014	SVM-MF	0.7515 ± 0.022	RF-MF	0.7355 ± 0.022	GRU-MF	0.7904 ± 0.012
LR-PCA	0.6890 ± 0.019	SVM-PCA	0.7741 ± 0.014	RF-PCA	0.7561 ± 0.025	GRU-PCA	0.8116 ± 0.007
LR-MissForest	0.7010 ± 0.018	SVM-MissForest	0.7779 ± 0.008	RF-MissForest	0.7890 ± 0.016	GRU-MissForest	0.8244 ± 0.012
						**Proposed GRU**-**D**	**0.8424 ± 0.012**

### Multi-task prediction on real datasets

In the reminder of the experiments, we use GRU-Simple as a representative for all GRU-Simple variant models^22–24^ since it obtains the best or comparable performance among them. The RNN models for multi-task learning with *m* tasks is almost the same as that for binary classification, except that 1) the soft-max prediction layer is replaced by a fully connected layer with *n* sigmoid logistic functions, and 2) a data-driven prior regularizer^43^, parameterized by comorbidity (co-occurrence) counts in training data, is applied to the prediction layer to improve the classification performance. We conduct multi-task classification experiments for *all 4 tasks* on PhysioNet and *20 ICD*-*9 code tasks* on MIMIC-III using all the GRU models. As shown in Table 2, the comparison of all methods are quite similar to that for mortality prediction task. GRU-D performs best in terms of average AUC score across all tasks and in most of the single tasks. On MIMIC-III dataset, GRU-MissForest and GRU-Simple provides the best performance among all baselines, while all simple imputations perform better than additional imputation baselines on PhysioNet dataset. Detailed results on each task can be found in supplementary information Section S4.

**Table 2:** Model performances measured by average AUC score (*mean* ± *std*) for multi-task predictions on real datasets.

Models	ICD-9 20 Tasks on MIMIC-III Dataset	All 4 Tasks on PhysioNet Dataset
GRU-Mean	0.7070 ± 0.001	0.8099 ± 0.011
GRU-Forward	0.7077 ± 0.001	0.8091 ± 0.008
GRU-Simple	0.7105 ± 0.001	0.8249 ± 0.010
GRU-CubicSpline	0.6372 ± 0.005	0.7451 ± 0.011
GRU-MICE	0.6717 ± 0.005	0.7955 ± 0.003
GRU-MF	0.6805 ± 0.004	0.7727 ± 0.003
GRU-PCA	0.7040 ± 0.002	0.8042 ± 0.006
GRU-MissForest	0.7115 ± 0.003	0.8076 ± 0.009
**Proposed GRU**-**D**	**0.7123** ± **0.003**	**0.8370** ± **0.012**

## Discussions

### Validating and interpreting the learned decays

To validate GRU-D model and demonstrate how it utilizes informative missing patterns, we take the PhysioNet mortality prediction as a study case, and show the input decay (*γ*_*x*_) plots and hidden decay weight (*W*_*γh*_) histograms for each input variable.

**Figure 5:**
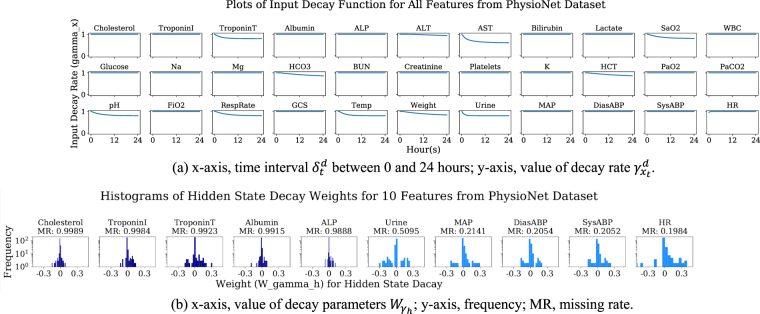
Plots of input decay *γ*_*xt*_ for all variables (top) and histrograms of hidden state decay weights ${W}_{{\gamma }_{h}}$ for 10 variables (bottom) in GRU-D model for predicting mortality on PhysioNet dataset.

From Fig. 5(a) which plots the learned input decay rate, we notice that the decay rate is almost constant for the majority of variables. However, a few variables have large decay which means that the value of the observation at the current time step is very important for prediction, and the model relies less on the previous observations. For example, the changes in the variable values of patient’s weight (missing rate 0.5452), arterial pH (missing rate 0.9118), temperature (missing rate 0.6915), and respiration rate (missing rate 0.8053) are known to impact the ICU patients health condition.

In Fig. 5(b), we plot the histograms of the hidden state decay parameters (weights *W*_*γh*_) corresponding to the input variables which has the highest (the 5 left subfigures) and the lowest (the 5 left subfigures) missing rates among all features. We find that the absolute parameter values are larger for variables with lower missing rate. For example, heart rate and cholesterol have the lowest and highest missing rates among all variables in this dataset. Our plot shows that hidden decay weights corresponding to heart rate have much larger scale than those of cholesterol. This indicates the time intervals of the variables with less missing rate have more impact on hidden state decay in our model. Notice that this is consistent with our preliminary investigation (in Fig. 1) that the mortality and the missing rate have larger correlations for variables with lower missing rate. These findings show that our model successfully recognise useful missing patterns from the data directly. The histograms for all variables is shown in the supplementary information Section S4.5.

### Early prediction capacity

**Figure 6:**
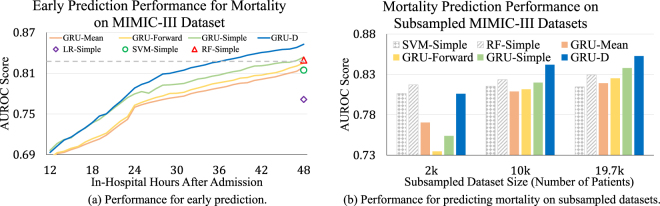
Early prediction capacity and model scalability comparisons of GRU-D and other RNN baselines on MIMIC-III dataset.

Although our model is trained on the first 48 hours data and makes prediction at the last time step, it can make predictions on the fly with partial observations. This is useful in applications such as health care, where early decision making is beneficial and critical for patient care. Figure 6(a) shows the online prediction results for MIMIC-III mortality task. We compared the RNN models with three widely used non-RNN models in practice, which are LR-Simple, SVM-Simple, and RF-Simple. Since these RNN models only take statistical mean from the training examples or use forward imputation on the time series, no future information of the time series is used when we make predictions at each time step for time series in the test dataset. As we can see, AUC score is around 0.7 at first 12 hours for all the GRU models and it keeps increasing with further observations. GRU-D and GRU-Simple, which explicitly handle missingness, perform consistently better than the other two RNN methods. In addition, GRU-D outperforms GRU-Simple when making predictions given time series of more than 24 hours, and has at least 2.5% higher AUC score after 30 hours. This indicates that GRU-D is able to capture and utilize long-range temporal missing patterns. Furthermore, GRU-D achieves similar prediction performance (i.e., same AUC) as best non-RNN baseline model with less time series data. As shown in the figure, GRU-D has same AUC performance at 36 hours as the best non-RNN baseline model (RF-Simple) at 48 hours. This 12 hour improvement of GRU-D over the two commonly used non-RNN baselines is highly significant in hospital settings such as ICU where accurate early prediction is necessary for making time-saving critical decisions.

### Model scalability with growing data size

In many practical applications with large datasets, model scalability is very important. To evaluate the model performance with different training dataset sizes, we subsample three smaller datasets of 2,000 and 10,000 admissions from the entire MIMIC-III dataset while keeping the same mortality rate. We compare our proposed models with all GRU baselines and two most competitive non-RNN baselines (SVM-Simple, RF-Simple) and present the prediction results in Fig. 6(b). We observe that all models can achieve improved performance given more training samples. However, the improvements of non-RNN baselines are quite limited compared to GRU models, and our GRU-D model achieves the best results on the two larger datasets. These results indicate the performance gap between GRU-D and non-RNN baselines will continue to grow as more data become available.

### Comparison to existing studies on mortality prediction

A series of work along the line of comparing and benchmarking the prediction performance of existing machine learning and deep learning models on MIMIC-III datasets have been conducted recently^44,45^. In the recent reproducibility summary^45^, the authors summarized the results of recently published methods for MIMIC-III mortality prediction task, and the results of our method is among the best as shown in the Table 4 of their paper. It is worth noting that there are no standard cohorts (i.e. no standard patient and variable inclusion criteria) in the MIMIC-III dataset for prediction analysis. The sample size and mortality rate are quite different among studies, and therefore the quantitative results are difficult to compare directly among all studies mentioned in the reproducibility summary. Our model outperformed the model with similar data settings^46^ by 1.27% AUROC score. To make fair and accurate comparison in our experiments, we choose the most competitive and relevant prediction baselines which are the RNN methods^22–24^. Similar to existing work^45^ which compared results across different cohorts using logistic regression and gradient boosting trees, we use logistic regression, SVM, and random forest as baseline prediction models and show relative improvement of 2.2% AUROC score on MIMIC-III dataset from our proposed models over the best of these baselines. In addition, to demonstrate the usefulness of modeling missing patterns, we show the results of all predictive methods which use the imputed data from various imputation approaches.

### Limitations

Our proposed model focused on the goal of making accurate and robust predictions on multi-variate time series data with missing values. This model relies on the information related to the prediction tasks, which is represented in the missing patterns, to improve the prediction performance over the original GRU-RNN baselines. If the missingness is not informative at all, or the inherent correlation between the missing patterns and the prediction tasks are not clear, our model may gain limited improvements or even fail. This requires a good understanding of the applied domains. Though our proposed model can be used in many health care applications and other application domains such as traffic and climate informatics, where the informative missingness presents, the decay mechanism needs to be explicitly designed.

The proposed method is not explicitly designed for filling in the missing values in the data, and can not be directly used in unsupervised settings without prediction labels. Though the proposed model’s structure can be modified for data imputation tasks, it requires additional evaluation and study, which is beyond the scope of this paper.

Our proposed models are only evaluated in retrospective observational study settings, which is due to the inherent limitation of the publicly available datasets used in our study. However, in general, the proposed model can be used in other applications in practice. First, we can deploy the proposed method in prospective observational study and validate the findings in the retrospective study. Second, by investigating the decay terms learnt from our model, doctors can assess the impact of missing data for each variable, and improve data collection strategies to acquire more important variables. In addition, the prediction from our model can be used as a surrogate to the scoring systems used in Intensive Care Unit (ICU), and thus it can be used to ensure similar baseline risks between comparative groups in clinical trials or to decide what kind of intervention needs to be given. Finally, the proposed model can also be used to study the real-time mortality risk assessment for ICU patients and can indicate how the health status of the patient evolves over time.

## Summary

Handling missing values in multivariate time series data using Recurrent Neural Networks is challenging. Off-the-shelf RNN architectures with imputation can only achieve comparable performance to Random Forests and SVMs, and moreover, they do not demonstrate the full advantage of representation learning. Using popular imputation methods leads to a time-consuming prediction procedure and may impair the prediction performance. To address the above issues, we propose a novel GRU-based model which captures the informative missingness by incorporating masking and time interval directly inside the GRU architecture. Our proposed GRU-D model with trainable decays has similar running time and space complexity to original RNN models, and are shown to provide promising performance and pull significantly ahead of non-deep learning methods on synthetic and real-world healthcare datasets. Although in our paper we focused on time-series data arising in intensive care units, we believe that our approaches will be widely useful for a variety of time-series prediction tasks arising in healthcare and beyond. In our future work, we will explore deep learning approaches to characterize missing-not-at-random data and we will conduct theoretical analysis to understand the behaviors of existing solutions for missing values.

## Electronic supplementary material

Supplementary information
