# Supplementary material for: Recurrent Neural Networks for Multivariate Time Series with Missing Values
Source: Sci Rep. 2018 Apr 17;8:6085. doi: 10.1038/s41598-018-24271-9 (PMC5904216; doi:10.1038/s41598-018-24271-9)
Supplement: Supplementary file 1 — Supplementary information [file 41598_2018_24271_MOESM1_ESM.pdf]

Supplementary Information for Recurrent Neural Networks for Multivariate Time Series with Missing Values

Zhengping Che[1], Sanjay Purushotham[1], Kyunghyun Cho[2], David Sontag[3], and Yan Liu[1]

[1]*Department of Computer Science, University of Southern California, Los Angeles, CA, USA 90089*
[2]*Department of Computer Science, New York University, New York, NY, USA 10012*
[3]*Department of Electrical Engineering and Computer Science, Massachusetts Institute of Technology, Cambridge, MA, USA 02139*

1 Investigation of relation between missingness and labels on two real health care datasets

In many time series applications, the pattern of missing variables in the time series is often informative and useful for prediction tasks. Here, we empirically confirm this claim on real health care dataset by investigating the correlation between the missingness and prediction labels (mortality and ICD-9 diagnosis categories). For each patient and its corresponding time series $\boldsymbol{X}$, we denote the missing rate for a variable d as $p^d_{\boldsymbol{X}}$ and calculate it by $p^d_{\boldsymbol{X}} = 1 - \frac{1}{T}\sum_{t=1}^{T} m^d_t$. Note that $p^d_{\boldsymbol{X}}$ is dependent on the mask vector (m^d_t) of that patient and the number of time steps T. Then for each patient, we will have a vector $p_{\boldsymbol{X}} = (p^1_{\boldsymbol{X}}, \ldots, p^D_{\boldsymbol{X}})^\top$ to represent the missing rates of all D time series features for that patient. For each prediction task denoted by label ℓ and each d-th feature, we compute the Pearson correlation coefficient between variable p^d and ℓ given the entire dataset. As shown in Figure 1(a), we observe that on MIMIC-III dataset the missing rates with low rate values are usually highly (either positive or negative) correlated with the labels. The distinct correlation between missingness and labels demonstrates usefulness of missingness patterns in solving prediction tasks. The list of variables and the list of the ICD-9 diagnosis categories can be found in Table 1 and 2 respectively. We can also find similar useful correlations in another real-world health care dataset, PhysioNet dataset, as shown in Figure 1(b), and the list of variables is shown in Table 3.

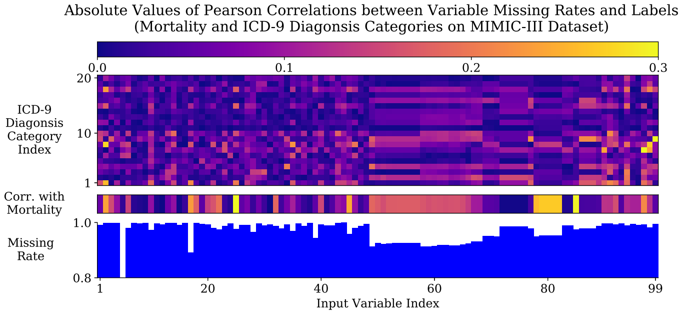

(a) The bottom figure shows the missing rate of each input variable. The middle figure shows the absolute values of Pearson correlation coefficients between missing rate of each variable and mortality. The top figure shows the absolute values of Pearson correlation coefficients between missing rate of each variable and each ICD-9 diagnosis category.

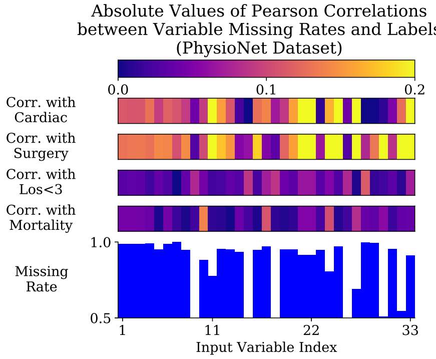

(b) The bottom figure shows the missing rate of each input variable. The top figures show the absolute values of Pearson correlation coefficients between missing rate of each variable and each of the 4 prediction tasks.

Figure 1: Demonstrations of informative missingness on two datasets. (color: absolute value of Pearson correlation coefficient.)

2 Model variations of GRU-D

A better model should have the flexibility to capture different missing patterns. In this section, we will discuss some variations of GRU-D model, and also compare some related RNN models which are used for time series with missing data with the proposed model. Empirical evaluations on these model variations will be provided later.

2.1 GRU model with different trainable decays

The proposed GRU-D applies trainable decays on both input and hidden state transitions in order to capture the temporal missing patterns explicitly, which is presented by Equation (1) and (2) below.

$$x_t^d \leftarrow m_t^d x_t^d + (1 - m_t^d)\gamma_{\boldsymbol{x}_t}^{\;d} x_{t'}^d + (1 - m_t^d)(1 - \gamma_{\boldsymbol{x}_t}^{\;d})\tilde{x}^d \tag{1}$$

$$\boldsymbol{h}_{t-1} \leftarrow \boldsymbol{\gamma}_{\boldsymbol{h}_t} \odot \boldsymbol{h}_{t-1}, \tag{2}$$

This decay idea can be straightforwardly generated to other parts inside the GRU models separately or jointly, given different assumptions on the impact of missingness. As comparisons, we also describe and evaluate several modifications of GRU-D model.

GRU-DI (Figure 2(a)) and **GRU-DS** (Figure 2(b)) decay only the input and only the hidden state by Equation (1) and (2), respectively. They can be considered as two simplified models of the proposed GRU-D. GRU-DI aims at capturing direct impact of missing values in the data, while GRU-DS captures more indirect impact of missingness. Another intuition comes from this perspective: if an input variable is just missing, we should pay more attention to this missingness; however, if an variable has been missing for a long time and keeps missing, the missingness becomes less important. We can utilize this assumption by decaying the masking. This brings us the model **GRU-DM** shown in Figure 2(c), where we replace the masking m_t^d fed into GRU-D in by

$$m_t^d \leftarrow m_t^d + (1 - m_t^d) \cdot \gamma_{\boldsymbol{m}_t}^{\;d} \cdot (1 - m_t^d) = m_t^d + (1 - m_t^d)\gamma_{\boldsymbol{m}_t}^{\;d}$$

where the equality holds since m_t^d is either 0 or 1. We decay the masking for each variable independently from others by constraining $\boldsymbol{W}_{\gamma_{\boldsymbol{m}}}$ to be diagonal.

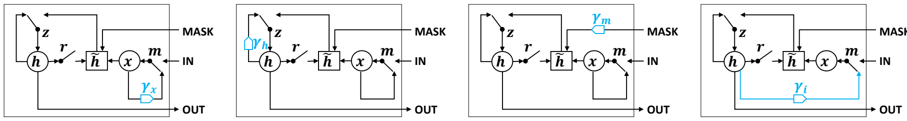

(a) GRU-DI (b) GRU-DS (c) GRU-DM (d) GRU-IMP

Figure 2: Graphical illustrations of variations of proposed GRU models.

2.2 GRU-IMP: Goal-oriented imputation model

We may alternatively let the GRU-RNN predict the missing values in the next timestep on its own. When missing values occur only during test time, we simply train the model to predict the measurement vector of the next time step as a language model and use it to fill the missing values during test time. This is unfortunately not applicable for some time series applications such as in health care domains, which also have missing data during training.

Instead, we propose goal-oriented imputation model here called **GRU-IMP**, and view missing values as latent variables in a probabilistic graphical model. Given a timeseries $\boldsymbol{X}$, we denote all the missing variables by $\mathcal{M}_{\boldsymbol{X}}$ and all the observed ones by $\mathcal{O}_{\boldsymbol{X}}$. Then, training a time-series classifier with missing variables becomes equivalent to maximizing the marginalized log-conditional probability of a correct label l, i.e., $\log p(l|\mathcal{O}_{\boldsymbol{X}})$.

The exact marginalized log-conditional probability is however intractable to compute, and we instead maximize its lowerbound:

$$\begin{aligned}\log p(l|\mathcal{O}_{\boldsymbol{X}}) &= \log \sum_{\mathcal{M}_{\boldsymbol{X}}} p\left(l|\mathcal{M}_{\boldsymbol{X}}, \mathcal{O}_{\boldsymbol{X}}\right) p\left(\mathcal{M}_{\boldsymbol{X}}|\mathcal{O}_{\boldsymbol{X}}\right) \\ &\geq \mathbb{E}_{\mathcal{M}_{\boldsymbol{X}} \sim p(\mathcal{M}_{\boldsymbol{X}}|\mathcal{O}_{\boldsymbol{X}})} \log p\left(l|\mathcal{M}_{\boldsymbol{X}}, \mathcal{O}_{\boldsymbol{X}}\right)\end{aligned}$$

where we assume the distribution over the missing variables at each time step is only conditioned on all the previous observations:

$$p\left(\mathcal{M}_{\boldsymbol{X}}|\mathcal{O}_{\boldsymbol{X}}\right) = \prod_{t=1}^{T} \prod_{1 \leq d \leq D}^{m_t^d=1} p(x_t^d|\boldsymbol{x}_{1:(t-1)}, \boldsymbol{m}_{1:(t-1)}, \boldsymbol{\delta}_{1:(t-1)})$$

Although this lowerbound is still intractable to compute exactly, we can approximate it by Monte Carlo method, which amounts to sampling the missing variables at each time as the RNN reads the input sequence from the beginning to the end, such that

$$x_t^d \leftarrow m_t^d x_t^d + (1 - m_t^d)\tilde{x}_t^d$$

where $\tilde{\boldsymbol{x}}_t \sim x_t^d|\boldsymbol{x}_{1:(t-1)}, \boldsymbol{m}_{1:(t-1)}, \boldsymbol{\delta}_{1:(t-1)}$.

By further assuming that $\tilde{\boldsymbol{x}}_t \sim \mathcal{N}\left(\boldsymbol{\mu}_t, \boldsymbol{\sigma}_t^2\right)$, $\boldsymbol{\mu}_t = \boldsymbol{\gamma}_t \odot (\boldsymbol{W}_x \boldsymbol{h}_{t-1} + \boldsymbol{b}_x)$ and $\boldsymbol{\sigma}_t = \mathbf{1}$, we can use a reparametrization technique widely used in stochastic variational inference to estimate the gradient of the lowerbound efficiently. During the test time, we simply use the mean of the missing variable, i.e., $\tilde{\boldsymbol{x}}_t = \boldsymbol{\mu}_t$, as we have not seen any improvement from Monte Carlo approximation in our preliminary experiments. We view this approach as a goal-oriented imputation method and show its structure in Figure 2(d). The whole model is trained to minimize the classification cross-entropy error ℓ_{log_loss} and we take the negative log likelihood of the observed values as a regularizer.

$$\ell = \ell_{log\_loss} + \lambda \frac{1}{N} \sum_{n=1}^{N} \frac{1}{T_n} \sum_{t=1}^{T_n} \frac{\sum_{d=1}^{D} m_t^d \cdot \log p(x_t^d|\mu_t^d, \sigma_t^d)}{\sum_{d=1}^{D} m_t^d}$$

3 Dataset preprocessing details

3.1 MIMIC-III datset preprocessing details

Here, we describe the preprocessing details for MIMIC-III dataset. MIMIC-III provides several relational database tables containing information of data relating to patients who stayed within the intensive care units (ICUs) at Beth Israel Deaconess Medical Center. The admission table contains over 58,000 hospital admission records of 38,645 adults and 7,875 neonates. We chose four tables namely inputevents-mv (fluids into patient, e.g. insulin), outputevents (fluids out of the patient, e.g. urine), labevents (lab test results, e,g. pH, Platelet count) and prescription events (drugs prescribed by doctors, e.g. aspirin and potassium chloride) to collect the patient data recorded in critical care units and hospital record systems. The inputevents-mv table collects the intake for patients monitored using the iMDSoft Metavision system. For our work, we use 19,714 admission records collected during 2008-2012 by Metavision data management system which is still employed at the hospital. The data collection and organization in Metavision system is much neater than the earlier Philips CareVue system [2001-2008]. From each of the four tables, we chose the top 50 items (i.e. features/variables) since these items are present in many of the patients' records. To avoid/reduce ambiguity and noisy observations, we ensured that all the measurements for a particular variable has only one unit of measurement. We also aggregated the multiple readings of a feature at a single time stamp based on the feature type. For instance, some inputevents features should be averaged while others need to be summed up. This resulted in 99 variables being extracted from the four tables for 19,714 patient admission records. The entire selected variable list and the variable index is shown in Table 1.

For each of the admission records, we collected both the variable value x_t and the time-stamp of observation s_t. In addition, for each admission record we queried the database tables to get the ICD-9 diagnosis codes. One admission record can be associated with multiple ICD-9 codes. We also queried the

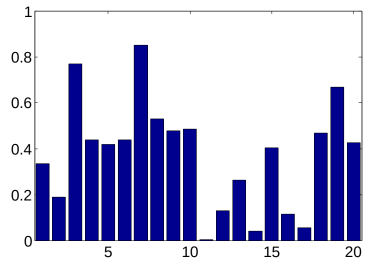

Figure 3: ICD-9 diagnosis class (category) distribution in MIMIC-III dataset. x-axis, ICD-9 diagnosis category id; y-axis, the ratio of admission records with the diagnosis code.

discharge time and death time from the Admissions table of MIMIC-III to find the mortality label for each admission record. The ICD-9 diagnosis code were grouped into 20 categories according to the information from the Thomson Reuters webpage, and the categories are shown in Table 2. The class distribution of the ICD-9 codes is shown in Figure 3.

3.2 PhysioNet dataset variable list

The original PhysioNet Challenge 2012 dataset consist of records with 42 variables. 6 of them are general descriptors collected on admission, and we only took the other 36 variables which are time series. Among the 36 variables, we further merged NIDiasABP, NIMAP, NISysABP to DiasABP, MAP, SysABP, respectively, and got 33 final variables. 11 of them are vital signs and 22 of them are lab measurements. The variables and their missing rates are shown in Table 3. We can find most vital signs have smaller missing rate than lab measurements.

3.3 Gesture synthetic dataset generation

The Gesture phase segmentation dataset is composed by features extracted from 7 videos with people gesticulating. It contains a time series with 18 numerical attributes and timestamps, and a set of 32 processed features with no timestamps. No missing values exist in this original dataset. Since the processed features are not exactly mapped to the timestamps, we use only the 18 raw features and their corresponding timestamps.

A phase label from 5 possible phases (rest, preparation, hold, stroke, and retraction) is assigned at each time step. Noticing that the labels for neighbouring time steps are usually the same, we first generated non-overlapped time series with same label from the original data, which has 9900 time stamps in total. From the beginning of each new phase, we truncate the time series by 30 time steps until the end of the phase segment. We ignored the last extracted time series if it is shorter than 7 time steps. After this step, we got 378 time series with different lengths. The numbers of time series of the 5 labels are $65, 115, 76, 49, 73$, respectively.

We then generated several synthetic datasets by manually introducing missing values. Our goal is that all the synthetic datasets have the same overall average missing rates (50%), while the correlations between the missing rates and the labels are different for all datasets. We did in the following ways to generate datasets with the desired properties. First, for each feature $d \in \{1, \cdots, 18\}$, we randomly sample a number $s_d \in \{-1, 1\}$ with equal probabilities to indicate whether the missing rate of that feature has positive or negative correlations with the labels. Then for each sample i, we randomly choose a missing rate $r_{i,d}$ from a uniform distribution $\mathcal{U}[0.3 + C \cdot s_d \cdot y_i, 0.7 + Cs \cdot_d \cdot y_i]$, where $y_i = \{1, \cdots, 5\}$ is the label for sample i, and C is a constant parameter for that synthetic dataset. We select a proper value of C to control the average absolute values of the Pearson correlation between missing rate for each feature r_d and the label y.

We then randomly introduce missing values based on the corresponding missing rate. We repeated the above steps to generate 4 synthetic datasets, with the average absolute correlation value to be $0, 0.2, 0.5, 0.8$.

3.4 Dataset statistics

For each of the three datasets used in our experiments, we list the number of samples, the number of input variables, the mean and max number of time steps for all the samples, and the mean of all the variable missing rates in Table 4.

Table 1: List of 99 extracted variables and their indexes after preprocessing MIMIC-III dataset.

**Table Name**	**Variable Names**
Output (15 Variables)	1. Gastric Gastric Tube, 2. Stool Out Stool, 3. Urine Out Incontinent, 4. Ultrafiltrate, 5. Foley, 6. Void, 7. Condom Cath, 8. Fecal Bag, 9. Ostomy (Output), 10. Chest Tube #1, 11. Chest Tube #2, 12. Jackson Pratt #1, 13. OR EBL, 14. Pre-Admission, 15. TF Residual
Input (33 Variables)	16. Albumin 5%, 17. Dextrose 5%, 18. Fresh Frozen Plasma, 19. Lorazepam (Ativan), 20. Calcium Gluconate, 21. Midazolam (Versed), 22. Phenylephrine, 23. Furosemide (Lasix), 24. Hydralazine, 25. Norepinephrine, 26. Magnesium Sulfate, 27. Nitroglycerin, 28. Insulin - Regular, 29. Insulin - Glargine, 30. Insulin - Humalog, 31. Heparin Sodium, 32. Morphine Sulfate, 33. Potassium Chloride, 34. Packed Red Blood Cells, 35. Gastric Meds, 36. D5 1/2NS, 37. LR, 38. K Phos, 39. Solution, 40. Sterile Water, 41. Metoprolol, 42. Piggyback, 43. OR Crystalloid Intake, 44. OR Cell Saver Intake, 45. PO Intake, 46. GT Flush, 47. KCL (Bolus), 48. Magnesium Sulfate (Bolus)
Lab Test (41 Variables)	49. Hematocrit, 50. White Blood Cells, 51. Platelet Count, 52. Hemoglobin, 53. MCHC, 54. MCH, 55. MCV, 56. Red Blood Cells, 57. RDW, 58. Potassium, 59. Sodium, 60. Chloride, 61. Bicarbonate, 62. Anion Gap, 63. Urea Nitrogen, 64. Creatinine, 65. Glucose, 66. Magnesium, 67. Total Calcium, 68. Phosphate, 69. INR(PT), 70. PT, 71. PTT, 72. Lymphocytes, 73. Monocytes, 74. Neutrophils, 75. Basophils, 76. Eosinophils, 77. Total Bilirubin, 78. PH, 79. Base Excess, 80. Calculated Total CO2, 81. PO2, 82. PCO2, 83. PH, 84. Specific Gravity, 85. Lactate, 86. Alanine Aminotransferase (ALT), 87. Asparate Aminotransferase (AST), 88. Alkaline Phosphatase, 89. Albumin
Prescription (10 Variables)	90. Aspirin, 91. Bisacodyl, 92. Docusate Sodium, 93. D5W, 94. Humulin-R Insulin, 95. Potassium Chloride, 96. Magnesium Sulfate, 97. Metoprolol Tartrate, 98. Sodium Chloride 0.9% Flush, 99. Pantoprazole

Table 2: Descriptions of MIMIC-III ICD-9 diagnoses categories.

**Task ID**	**ICD-9 Codes**	**Diagnoses Groups**
1	001 - 139	Infectious and Parasitic Diseases
2	140 - 239	Neoplasms
3	240 - 279	Endocrine, Nutritional, Metabolic, Immunity
4	280 - 289	Blood and Blood-Forming Organs
5	290 - 319	Mental Disorders
6	320 - 389	Nervous System and Sense Organs
7	390 - 459	Circulatory System
8	460 - 519	Respiratory System
9	520 - 579	Digestive System
10	580 - 629	Genitourinary System
11	630 - 677	Pregnancy, Childbirth, and the Puerperium
12	680 - 709	Skin and Subcutaneous Tissue
13	710 - 739	Musculoskeletal System and Connective Tissue
14	740 - 759	Congenital Anomalies
15	780 - 789	Symptoms
16	790 - 796	Nonspecific Abnormal Findings
17	797 - 799	Ill-defined and Unknown Causes of Morbidity and Mortality
18	800 - 999	Injury and Poisoning
19	V Codes	Supplemental V-Codes
20	E Codes	Supplemental E-Codes

Table 3: List of 33 variables after preprocessing PhysioNet dataset.

**Index**	**Variable Name**	**Category**	**Missing Rate**
1	ALP	Lab Measurement	0.9888
2	ALT	Lab Measurement	0.9885
3	AST	Lab Measurement	0.9885
4	Albumin	Lab Measurement	0.9915
5	BUN	Lab Measurement	0.9496
6	Bilirubin	Lab Measurement	0.9884
7	Cholesterol	Lab Measurement	0.9989
8	Creatinine	Lab Measurement	0.9493
9	(NI)DiasABP	Vital Sign	0.2054
10	FiO2	Vital Sign	0.8830
11	GCS	Vital Sign	0.7767
12	Glucose	Lab Measurement	0.9528
13	HCO3	Lab Measurement	0.9507
14	HCT	Lab Measurement	0.9338
15	HR	Vital Sign	0.1984
16	K	Lab Measurement	0.9477
17	Lactate	Lab Measurement	0.9709
18	(NI)MAP	Vital Sign	0.2141
19	Mg	Lab Measurement	0.9507
20	Na	Lab Measurement	0.9508
21	PaCO2	Vital Sign	0.9157
22	PaO2	Vital Sign	0.9158
23	Platelets	Lab Measurement	0.9489
24	Resp Rate	Vital Sign	0.8053
25	SaO2	Lab Measurement	0.9705
26	(NI)SysABP	Vital Sign	0.2052
27	Temp	Vital Sign	0.6915
28	Troponin-I	Lab Measurement	0.9984
29	Troponin-T	Lab Measurement	0.9923
30	Urine	Vital Sign	0.5095
31	WBC	Lab Measurement	0.9532
32	Weight	Lab Measurement	0.5452
33	pH	Lab Measurement	0.9118

Table 4: Details about the three datasets used in our experiments.

	**MIMIC-III**	**PhysioNet**	**Gesture**
# of samples (N)	$19,714$	$4,000$	378
# of variables (D)	99	33	18
Mean of # of time steps	35.89	68.91	21.42
Maximum of # of time steps	150	155	30
Mean of variable missing rate	0.9621	0.8225	N/A

4 Supplemental experiments and discussions

4.1 GRU model size comparison

We show the the statistics of our GRU based models for three datasets in Table 5. For the two real datasets, we show the numbers for mortality prediction, and the numbers for multi-task classifications are also close for all the compared models.

Table 5: Size comparison of GRU models used in our experiments. *Vars.*: all input features/variables in that dataset; *Size*: number of hidden states ($\boldsymbol{h}$) in GRU; *Pars.*: all parameters in the neural network model.

		Other GRU Models	GRU-Simple	GRU-D
**Gesture**	# of *Vars.*	18	18	18
	Size	64	50	55
	# of *Pars.*	16,281	16,025	16,561
**MIMIC-III**	# of *Vars.*	99	99	99
	Size	100	56	67
	# of *Pars.*	60,105	59,533	60,436
**PhysioNet**	# of *Vars.*	33	33	33
	Size	64	43	49
	# of *Pars.*	18,885	18,495	18,838

4.2 Multi-task prediction results

The AUC scores for predicting each of the 4 tasks on PhysioNet dataset are shown in Figure 4, and those for each of the 20 ICD-9 diagnosis categories on MIMIC-III dataset are shown in Figure 5. The proposed GRU-D achieves the best average AUC score on both datasets, and it wins 2 most challenging tasks on PhysioNet dataset and 11 of the 20 ICD-9 prediction tasks on MIMIC-III dataset.

4.3 Evaluation on multi-layer RNNs

We also conducted experiments on 2-layer RNN models to demonstrate the superiority of our proposed GRU-D models can be generalized to multi-layer RNNs. For all baseline and proposed GRU models, we add one standard GRU layer on top of the baseline or proposed GRU layer. We tested models both with similar number of parameters to single layer models and with more parameters. As shown in Table 6 and 7, our GRU-D model consistently outperforms other baselines in all cases, and models with moderate size perform as good as larger

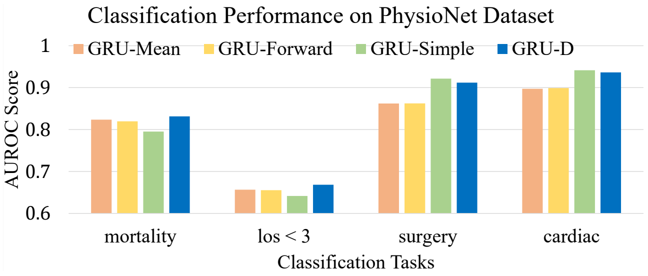

Figure 4: Performance for predicting all 4 tasks on PhysioNet dataset. *mortality*, in-hospital mortality; *los*<3, length-of-stay less than 3 days; *surgery*, whether the patient was recovering from surgery; *cardiac*, whether the patient had a cardiac condition.

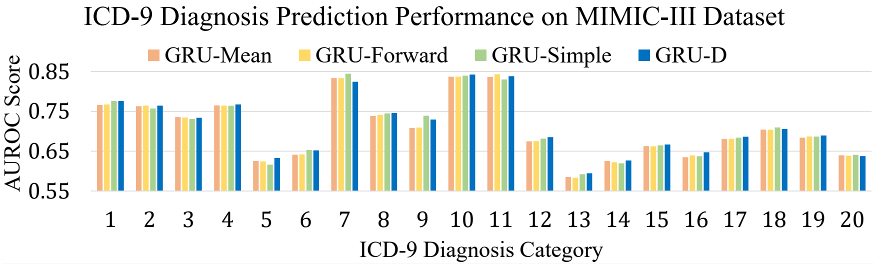

Figure 5: Performance for predicting 20 ICD-9 diagnosis categories on MIMIC-III dataset.

models with more parameters. Compared with 1-layer RNNs, all models with deeper structures perform much better on the larger MIMIC-III dataset but no better on the relatively smaller PhysioNet dataset.

4.4 Empirical comparison of GRU-D model variations

As a thorough empirical comparison, we test all GRU model variations mentioned in supplementary information Section 2 along with the proposed GRU-D, which are 4 models with trainable decays (GRU-DI, GRU-DS, GRU-DM, GRU-IMP). The results on three versions of GRU-simple models are also compared in the table. The results are shown in Table 8. As we can see, GRU-D performs best among these models.

4.5 Histograms of hidden state decay weights

We show the histograms of the hidden state decay weights for all 33 variables in figure 6. The variables are sorted by their missing rate in this dataset, e.g., the variable with highest missing rate (Cholesterol) is shown on top-left, and that with lowest missing rate (HR) is on bottom-right. We can find on average,

Table 6: Comparison of multi-layer GRU models for mortality prediction on PhysioNet dataset. *Size*: numbers of hidden states ($\boldsymbol{h}$) of the two GRU layers. *Pars.*: all parameters in the neural network model.

Models		PhysioNet		
		Size	# of *Pars.*	AUC score
Similar size	GRU-Mean	40, 40	18, 643	0.8157 ± 0.008
	GRU-Forward	40, 40	18, 643	0.8205 ± 0.008
	GRU-Simple	32, 32	18, 947	0.8159 ± 0.007
	**GRU-D**	34, 34	18, 599	$\mathbf{0.8420 \pm 0.009}$
Larger size	GRU-Mean	64, 64	43, 651	0.8199 ± 0.002
	GRU-Forward	64, 64	43, 651	0.8112 ± 0.035
	GRU-Simple	43, 64	39, 250	0.8208 ± 0.009
	**GRU-D**	49, 64	40, 739	$\mathbf{0.8363 \pm 0.013}$

the (absolute) weight values for low missing rate variable is larger, implies the importance of the missingness of those variables in our model.

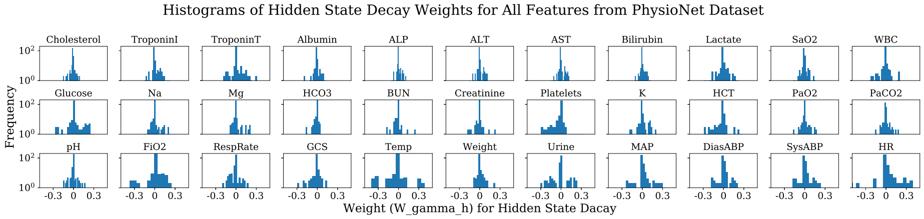

Figure 6: Histrograms of hidden state decay weights $\boldsymbol{W}_{\gamma_{\boldsymbol{h}}}$ for all variables (bottom) in GRU-D model for predicting mortality on PhysioNet dataset. x-axis, value of decay parameter $\boldsymbol{W}_{\gamma_{\boldsymbol{h}}}$; y-axis, frequency. Variables are sorted in descending order of missing rate.

Table 7: Comparison of multi-layer GRU models for mortality prediction on MIMIC-III dataset. *Size*: numbers of hidden states ($\boldsymbol{h}$) of the two GRU layers. *Pars.*: all parameters in the neural network model.

Models		MIMIC-III		
		Size	# of *Pars.*	AUC score
Similar size	GRU-Mean	$66, 66$	$59, 271$	0.9538 ± 0.005
	GRU-Forward	$66, 66$	$59, 271$	0.9441 ± 0.005
	GRU-Simple	$46, 46$	$60, 355$	0.9527 ± 0.005
	**GRU-D**	$52, 52$	$60, 989$	$\mathbf{0.9606 \pm 0.002}$
Larger size	GRU-Mean	$100, 128$	$148, 067$	0.9539 ± 0.006
	GRU-Forward	$100, 128$	$148, 067$	0.9457 ± 0.005
	GRU-Simple	$56, 128$	$130, 643$	0.9523 ± 0.003
	**GRU-D**	$67, 128$	$135, 759$	$\mathbf{0.9618 \pm 0.002}$

Table 8: Model performances of GRU variations measured by AUC score ($mean \pm std$) for mortality prediction.

Models		MIMIC-III	PhysioNet
Baselines	GRU-simple w/o δ	0.8367 ± 0.009	0.8226 ± 0.010
	GRU-simple w/o m	0.8266 ± 0.009	0.8125 ± 0.005
	**GRU-simple**	0.8380 ± 0.008	0.8155 ± 0.004
Proposed	GRU-DI	0.8345 ± 0.006	0.8328 ± 0.008
	GRU-DS	0.8425 ± 0.006	0.8241 ± 0.009
	GRU-DM	0.8342 ± 0.005	0.8248 ± 0.009
	GRU-IMP	0.8248 ± 0.010	0.8231 ± 0.005
	**GRU-D**	$\mathbf{0.8527 \pm 0.003}$	$\mathbf{0.8424 \pm 0.012}$
